# A global database on coral recovery following marine heatwaves

**DOI:** 10.1038/s41597-024-03221-3

**Published:** 2024-04-11

**Authors:** Robert van Woesik, Chelsey Kratochwill

**Affiliations:** Institute for Global Ecology, Florida Institute of Technology, Melbourne, Florida 32901 United States of America

**Keywords:** Climate-change ecology, Marine biology

## Abstract

Coral reefs support the world’s most diverse marine ecosystem and provide invaluable goods and services for millions of people worldwide. They are however experiencing frequent and intensive marine heatwaves that are causing coral bleaching and mortality. Coarse-grained climate models predict that few coral reefs will survive the 3 °C sea-surface temperature rise in the coming century. Yet, field studies show localized pockets of coral survival and recovery even under high-temperature conditions. Quantifying recovery from marine heatwaves is central to making accurate predictions of coral-reef trajectories into the near future. Here we introduce the world’s most comprehensive database on coral recovery following marine heatwaves and other disturbances, called Heatwaves and Coral-Recovery Database (HeatCRD) encompassing 29,205 data records spanning 44 years from 12,266 sites, 83 countries, and 160 data sources. These data provide essential information to coral-reef scientists and managers to best guide coral-reef conservation efforts at both local and regional scales.

## Background & Summary

The intensity and frequency of anomalously high ocean temperatures have increased over the past four decades^1,2^. Such marine heatwaves have been particularly evident on coral reefs, globally^3–5^. High ocean temperatures lead to coral bleaching, coral mortality, and changes in coral assemblages. Many recent studies have focused on coral bleaching as the immediate effect of heatwaves at oceanic scales^4–6^ but only a few studies (see for example Gonzalez-Barrios *et al*.^7^) have focused on coral recovery. Likewise, several recent databases have addressed oceanic scale coral bleaching^8^ and coral cover^9–11^ that complement the work presented in this paper. The dynamics and trajectories of corals are dependent on a suite of parameters, including the intensity of the heatwave, the geographical location and depth of the site, how much coral remains following the heatwave, the composition of the community, how quickly the corals grow, and the extent of subsequent recruitment following the heatwave. Heatwaves and Coral-Recovery Database (HeatCRD)^1^ fulfills an urgent need to compile data following marine heatwaves at oceanic scales to determine (i) how rapidly coral reefs recover from marine heatwaves, (ii) to what extent recovery varies geographically^12^, and (iii) which local conditions influence recovery rates?

**Fig. 1:**
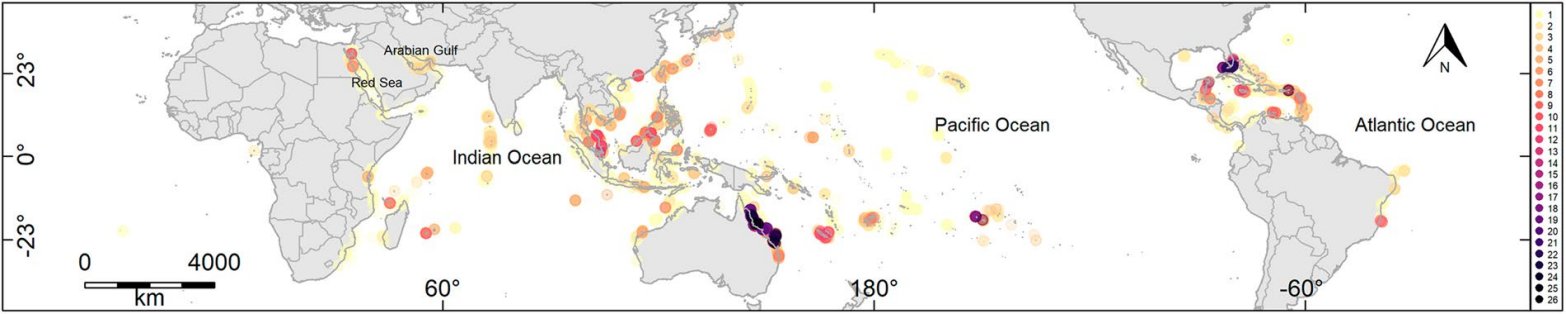
The global HeatCRD (Heatwaves and Coral-Recovery Database) study sites (n = 12,266)^1^. The colors signify the number of repeated surveys (from 1 – 26) at each study site, from 83 countries from 1977 to 2020.

Most field studies on coral reefs estimate the percentage of coral cover, which is the two-dimensional coverage that corals occupy across a coral-reef substrate. The primary data presented here is the percentage of total coral cover at a study site over time. A study site is a unique latitude-longitude coordinate point at a given depth. To date, we have 29,205 data records for 12,266 sites, over 4 decades from 1977 to 2020 (Fig. 1, Supplementary Table 1)^1^. There are two main data sources in the database, the first being a compilation of data from established monitoring programs (73%), and the second being new data extracted from the literature (27%). All time-series datasets have been checked at multiple levels and are quality-controlled. The database also contains environmental variables at each site, including site exposure to waves, distance to land, level of protection from fishing, habitat type, mean turbidity, and a suite of sea-surface temperature metrics at the time of the survey.

## Methods

**Fig. 2:**
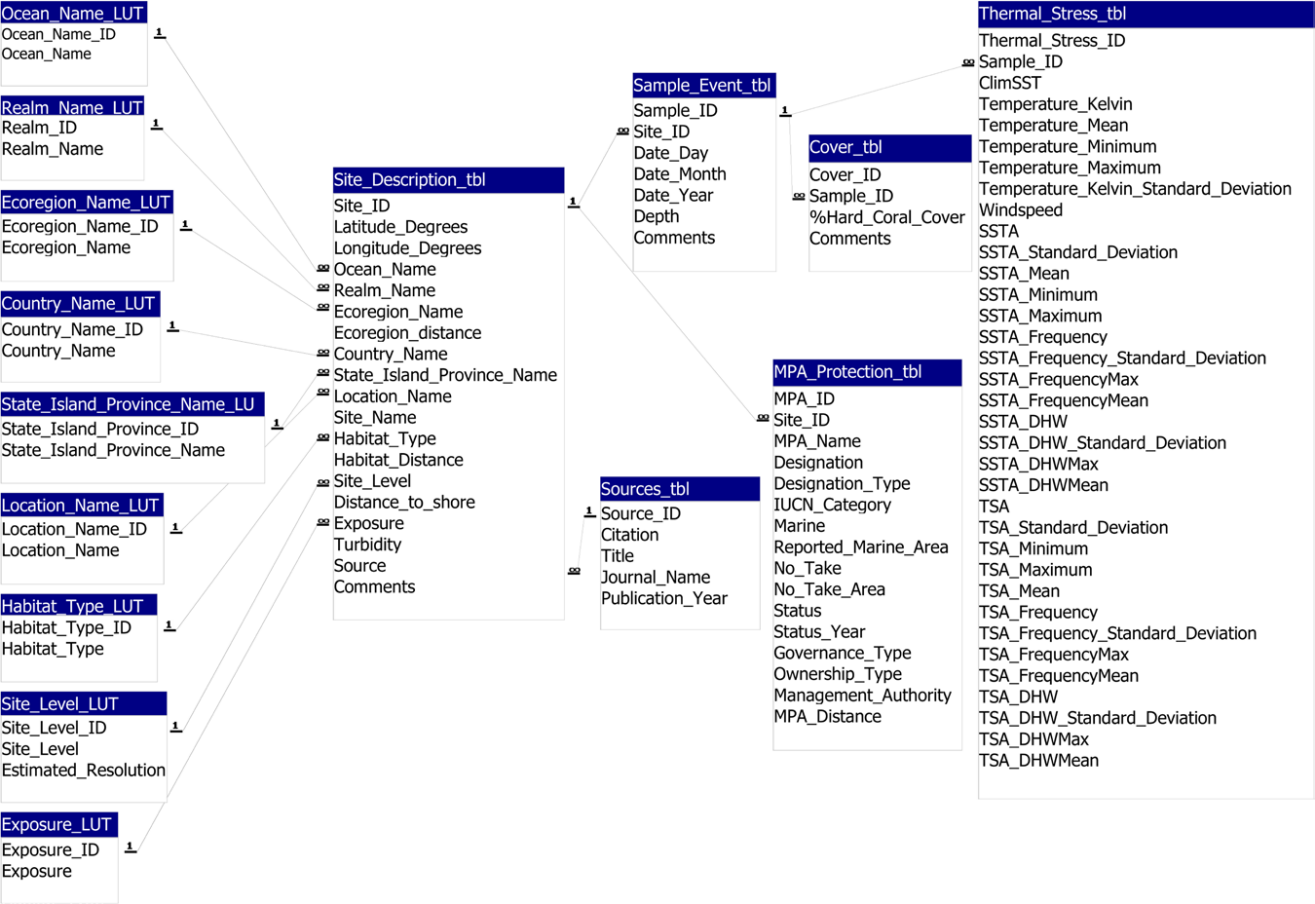
Schematic of the global HeatCRD (Heatwaves and Coral-Recovery Database)^1^ showing the relationships among the 15 Tables.

To date, we have coral data for 12,266 sites, from 83 countries, from 1977 to 2020 (Fig. 1; Supplementary Table 1)^1^. The Heatwaves and Coral Recovery Database (HeatCRD) is available as a Microsoft Access database file and as a SQLite database file^1^, the latter of which is directly accessible through R. Examples of the R code that extract data from the SQLite files, which are ready for data analysis, are provided as “DB_Querying_RSQLite.R”. Data in the HeatCRD are stored in 15 related Tables (see Fig. 2, Schematic of the database structure). Some geographical regions have more comprehensive time-series data than other regions, for example, the Great Barrier Reef, in Australia, and the Florida Keys, USA, have the most comprehensive time-series data (Fig. 1).

The primary geographical variable in the HeatCRD is a ‘site’ on a reef, recorded as a latitude and longitude coordinate. The static locality data (i.e., latitudinal and longitudinal coordinates, distance to land, and exposure) are stored in the Table “Site_Description_tbl”. A site can have multiple sampling events (Fig. 1) (i.e., multiple depths and/or multiple dates sampled), and these temporal events are stored separately in the Table “Sample_Event_tbl”. Data corresponding to these sampling events are stored in two related Tables: “Cover_tbl” (% hard coral cover) and “Thermal_Stress_tbl”. Tables with enumerated lists are used to ensure integrity in naming conventions — such Tables are denoted with “LUT” (look-up-table). Data in the HeatCRD are stored in 15 related Tables (Fig. 2).

Coral-cover data were extracted from the primary literature using WebPlotDigitizer^13^. Sampling points that fell on land or were >1 km from any coral reef were removed. If sites were not named or given explicit coordinates, the coordinates were estimated and a comment was added to the data table. The coordinates were entered into Google Earth and the location names, distance to land in meters, and exposure were determined and recorded for each site. Exposure to waves was based on a site’s potential exposure to predominate winds, swell, and fetch (i.e., the extent of open ocean). Mean turbidity (Kd_490_) was added for each site^14^; although turbidity is technically the suspended particles in the water column, K_d_ represents changes in water clarity from both particles, dissolved materials, and the water itself. We used the term turbidity in previous publications and therefore we will remain consistent in the present study. The Marine Ecoregions of the World (MEOW) shapefiles^15^ and IUCN’s (International Union for Conservation of Nature) World Database on Protected Areas^16^ were used to determine in which marine realm and protected area each site was located. Veron’s ecoregions^17^ shapefiles were used to determine the ecoregion of each site. Data on the types of reef habitats were extracted from the Allen Coral Atlas^18^.

### Normalization

If the site coordinates were not already in decimal degrees in the original data, they were converted to decimal degrees in the HeatCRD. Latitude and longitude coordinates were determined with Google Earth when coordinate information was not explicitly provided in the text of the published papers. The Coral Reef Temperature Anomaly Database (CoRTAD version 6)^19^, which is a collection of sea surface temperature variables, was used to extract temperature metrics for each sampling event. CoRTAD values were only extracted for a sampling event if sampled data had a clearly defined month and year — where sampling events were missing a date, the 15^th^ day of the month was used. For any data given as a range (i.e., depth or date), the midpoint was taken and a comment was added to the HeatCRD.

## Data Records

The dataset is available at Figshare^1^. Below we describe 15 Tables (also see Fig. 2) that comprise the HeatCRD (where LUT stands for “lookup table”):

1. Site_Description_tbl,
2. Sample_Event_tbl,
3. Cover_tbl,
4. MPA_Protection_tbl,
5. Thermal_Stress_tbl,
6. Sources_tbl,
7. Country_Name_LUT,
8. Realm_Name_LUT,
9. Ecoregion_Name_LUT,
10. Exposure_LUT,
11. Habitat_Type_LUT,
12. Location_Name_LUT.
13. Ocean_Name_LUT,
14. Site_Level_LUT, and
15. State_Island_Province_Name_LUT

1. Site Description (Site_Description_tbl)
  Latitude_Degrees: latitude coordinates in decimal degrees.
  Longitude_Degrees: longitude coordinates in decimal degrees.
  Ocean_Name: the ocean where sampling took place.
  Realm_Name: the marine realm where sampling took place^15^.
  Ecoregion_Name: the ecoregion where sampling took place^17^.
  Ecoregion_distance: distance in degrees of the site from the nearest ecoregion polygon.
  Country_Name: the country where sampling took place.
  State_Province_Island_Name: the state, province, or island where sampling took place.
  Location_Name: site or reef where sampling took place.
  Site_Name: the accepted name of the site or the name given by the team that sampled the reef.
  Habitat_Type: Habitat type where sampling took place^18^.
  Site Level: the specificity of coordinates of the sampling site.
  Distance_to_Shore: the distance (m) of the sampling site from the nearest land.
  Exposure: a site was considered exposed if it had > 20 km of fetch, if there were strong seasonal winds, or if the site faced the prevailing winds. Otherwise, the site was considered sheltered.
  Turbidity: 490_kd_ with a buffer of 100 m^14^.
  Source: the original source of the data.
  Comments: Comments on any issues with the site or additional information.
2. Sample Event Information (Sample_Event_tbl)
  Site_ID: site ID field from Site_Description_tbl.
  Date_Day: the day of the sampling event.
  Date_Month: the month of the sampling event.
  Date_Year: the year of the sampling event.
  Depth: depth (m) of the sampling site.
  Comments: comments on any issue or additional information about the sampling event.
3. Benthic Cover Information (Cover_tbl)
  Sample_ID: sample ID field from Sample_Event_tbl.
  Percent_Hard_Coral_Cover: percentage live coral cover.
  Percent_Macroalgal_Cover: percentage of macroalgal cover.
  Comments: comments on any issue or additional information on benthic cover.
4. Site Protection Information (MPA_Protection_tbl)
  Site_ID: site ID field from Site_Description_tbl.
  MPA_Name: name of the protected area^16^.
  Designation: designation of the protected area^16^.
  Designation_Type: category of the protected area^16^.
  IUCN_Category: IUCN management category^16^.
  Marine: describes if a protected area is totally or partially within the marine habitat^16^.
  Reported_Marine_Area: area in km^2^ of protected area in marine habitat^16^.
  No_Take: whether the taking of resources is prohibited^16^.
  No_Take_Area: the area in km^2^ of no take^16^.
  Status: status of the protected area^16^.
  Status_Year: the year the status of the protected area was effective^16^.
  Governance_Type: organization/government in charge of the protected area^16^.
  Ownership_Type: organization/government that legally ‘owns’ a protected area^16^.
  Management_Authority: group that manages the protected area^16^.
  Distance_to_MPA: distance of site to nearest MPA polygon in degrees^16^.
5. Environmental Information (Thermal_Stress_tbl)
  Sample_ID: sample ID field from Sample_Event_tbl.
  ClimSST: CoRTAD^19^. [Climatological Sea-Surface Temperature (SST)] based on weekly SSTs for the study time frame, created using a harmonics approach.
  Temperature_ Kelvin: CoRTAD^19^. SST in Kelvin.
  Temperature_Mean: CoRTAD^19^. Mean SST in degrees Celsius.
  Temperature_Minimum: CoRTAD^19^. Minimum SST in degrees Celsius.
  Temperature_Maximum: CoRTAD^19^. Maximum SST in degrees Celsius.
  Temperature_Kelvin_Standard_Deviation: CoRTAD^19^. The standard deviation of SST in Kelvin.
  Windspeed: CoRTAD^19^. Weekly-averaged 10 m wind speed time series from 1982–2012. Units are in meters per hour.
  SSTA: CoRTAD^19^. (Sea-Surface Temperature Anomaly) weekly SST minus weekly climatological SST.
  SSTA_Standard_Deviation: CoRTAD^19^. The Standard Deviation of weekly SSTA in degrees Celsius over the entire period.
  SSTA_Mean: CoRTAD^19^. The mean SSTA in degrees Celsius over the entire period.
  SSTA_Minimum: CoRTAD^19^. The minimum SSTA is in degrees Celsius over the entire period.
  SSTA_Maximum: CoRTAD^19^. The maximum SSTA is in degrees Celsius over the entire period.
  SSTA_Frequency: CoRTAD^19^. (Sea Surface Temperature Anomaly Frequency) Number of times over the previous 52 weeks that SSTA >  = 1 degree Celsius.
  SSTA_Frequency_Standard_Deviation: CoRTAD^19^. The standard deviation of SSTA Frequency in degrees Celsius over the entire period of 23 years.
  SSTA_FrequencyMax: CoRTAD^19^. The maximum SSTA Frequency is in degrees Celsius over the entire period.
  SSTA_FrequencyMean: CoRTAD^19^. The mean SSTA Frequency is in degrees Celsius over the entire period of 23 years.
  SSTA_DHW: CoRTAD^19^. (Sea Surface Temperature Degree Heating Weeks) the sum of the previous 12 weeks when SSTA >  = 1 degree Celsius.
  SSTA_DHW_Standard_Deviation: CoRTAD^19^. The standard deviation SSTA DHW in degrees Celsius over the entire period.
  SSTA_DHWMax: CoRTAD^19^. The maximum SSTA DHW in degrees Celsius over the entire period of 23 years.
  SSTA_DHWMean: CoRTAD^19^. The mean SSTA DHW in degrees Celsius over the entire period of 23 years.
  TSA: CoRTAD^19^. (Thermal Stress Anomaly) weekly SST minus the maximum of weekly climatological SSTs in degrees Celsius.
  TSA_Standard_Deviation: CoRTAD^19^. The standard deviation of TSA in degrees Celsius over the entire period of 23 years.
  TSA_Minimum: CoRTAD^19^. The minimum TSA is in degrees Celsius over the entire period of 23 years.
  TSA_Maximum: CoRTAD^19^. The maximum TSA in degrees Celsius over the entire period of 23 years.
  TSA_Mean: CoRTAD^19^. The mean TSA in degrees Celsius over the entire period of 23 years.
  TSA_Frequency: CoRTAD^19^. The number of times over the previous 52 weeks that TSA >  = 1 degree Celsius.
  TSA_Frequency_Standard_Deviation: CoRTAD^19^. The standard deviation of the frequency of TSA in degrees Celsius over the entire period of 23 years.
  TSA_FrequencyMax: CoRTAD^19^. The maximum TSA frequency in degrees Celsius over the entire period of 23 years.
  TSA_FrequencyMean: CoRTAD^19^. The mean TSA frequency in degrees Celsius over the entire period of 23 years.
  TSA_DHW: CoRTAD^19^. (Thermal Stress Anomaly Degree Heating Weeks) the sum of the previous 12 weeks when TSA >  = 1 degree Celsius.
  TSA_DHW_Standard_Deviation: CoRTAD^19^. The standard deviation of TSA DHW in degrees Celsius over the entire period of 23 years.
  TSA_DHWMax: CoRTAD^19^. The maximum TSA DHW in degrees Celsius over the entire period of 23 years.
  TSA_DHWMean: CoRTAD^19^. The mean TSA DHW in degrees Celsius over the entire period of 23 years.
6. Source Information (Sources_tbl)
  Citation: source citations with author name and year published.
  Title: title of publication.
  Journal_Name: name of publication journal.
  Publication_Year: year of publication.
7. Country Names (Country_Name_LUT)
  Country_Name: name of the country where sampling took place.
8. Realm Names (Realm_Name_LUT)
  Realm_Name: name of the marine realm where sampling took place^15^.
9. Ecoregion Information (Ecoregion_Name_LUT):
  Ecoregion_Name: name of ecoregion of site location^17^.
10. Exposure Type (Exposure_LUT)
  Exposure: type of wave exposure at the site.
11. Habitat Information (Habitat_Type_LUT)
  Habitat_Type: name of habitat at the sampling location^18^.
12. Location Information (Location_Name_LUT)
  Location_Name: the island, group, or reef where sampling took place.
13. Ocean Information (Ocean_Name_LUT)
  Ocean_Name: name of ocean.
14. Site-level Information (Site_Level_LUT)
  Site_Level: Level of specificity of a site’s coordinates, ranging from an exact coordinate to a regional estimate.
  Estimated_Resolution: estimation of resolution of site.
15. State, Island, or Province information (State_Province_Island_Name_LUT)

State_Island_Province_Name: state, island, or province of site.

## Database Queries

Fourteen summary queries have been created to help visualize and organize the HeatCRD data. The queries include:

1. Query_1_All_Sites,
2. Query_2_All_Samples,
3. Query_3_All_Cover,
4. Query_4_All_Cover_All_Variables,
5. Query_5_Time_Series_Years,
6. Query_6_Time_Series_Sites,
7. Query_7_Time_Series_Samples,
8. Query_8_Time_Series_Cover,
9. Query_9_Time_Series_All_Variables,
10. Query_10_Samples_by_Ocean,
11. Query_11_Samples_by_Source,
12. Query_12_Sites_Date_Count,
13. Query_13_Time_Series_Samples_by_Ocean, and
14. Query_14_Time_Series_Samples_by_Source.

## Technical Validation

The HeatCRD was curated by a Database Administrator (Chelsey Kratochwill). Other contributions were made by Amie Stanley and Trinity DiNunzio. Coral-cover datasets were created using WebPlotDigitizer^13^ to extract information from the literature. When coral-cover datasets were added, there was a four-point procedure to validate and standardize the site locations, including the following:

1. To ensure consistency in the naming of site locations, the latitude and longitude coordinates were entered into Google Earth. The country and location names were all cross-checked and verified.
2. All latitude and longitude coordinates were compared to ensure that a sampling event was not duplicated across multiple dataset sources.
3. Coordinate points were removed if they: (i) were erroneous (i.e., a coordinate point was negative when it should be positive), (ii) occurred on land, or (iii) were >1 km from a coral reef.
4. Environmental and site data were added to each site, which included reef exposure, distance to the nearest shoreline (m), habitat, ecoregion, MPA, and CoRTAD environmental variables.

### Supplementary information

Supplementary Table 1

## Data Availability

All R code that was used in the HeatCRD is provided.
