# Supplementary material for: A global database on coral recovery following marine heatwaves
Source: Sci Data. 2024 Apr 11;11:367. doi: 10.1038/s41597-024-03221-3 (PMC11009248; doi:10.1038/s41597-024-03221-3)
Supplement: Supplementary file 1 — Supplementary Table 1 [file 41597_2024_3221_MOESM1_ESM.pdf]

Supplementary Table

A global database on coral recovery following marine heatwaves

Robert van Woesik[1*] and Chelsey Kratochwill[1]

[1]Institute for Global Ecology, Florida Institute of Technology, Melbourne, Florida, 32901, United States of America; *Corresponding author: R. van Woesik (rvw@fit.edu)

Supplementary Table 1. Description of data sources in the HeatCRD (Heatwaves and Coral Recovery Database).

Oceans	Realms	Time-period	Depth Range (m)	Number of Samples	Year Published	Source	Source Type
Pacific	Central Indo-Pacific	2011-2019	4	47	2023	Abesamis et al. 2023	Primary Literature
Pacific	Eastern Indo-Pacific	1992-2002	10	77	2005	Adjeroud et al. 2005	Primary Literature
Pacific	Eastern Indo-Pacific	1991-2006	N/A	16	2009	Adjeroud et al. 2009	Primary Literature
Red Sea	Western Indo-Pacific	2015	1.8-7.6	22	2021	Aeby et al. 2021	Primary Literature
Indian	Western Indo-Pacific	2010-2014	4	5	2015	Aldyza et al. 2015	Primary Literature
Atlantic	Tropical Atlantic	2010-2012	N/A	8	2014	Alemu I and Clement 2014	Primary Literature
Pacific	Tropical Eastern Pacific	2003	N/A	10	2005	Alvarado et al. 2005	Primary Literature
Atlantic	Tropical Atlantic	1997-2016	13.5	52	2022	Alves et al. 2022	Primary Literature

Indian	Western Indo-Pacific	2009	10-20	11	2011	Amri 2011	Primary Literature
Pacific	Eastern Indo-Pacific	2004	0.8-2.4	11	2004	Andrews 2004	Primary Literature
Pacific	Central Indo-Pacific	1998	10	8	2001	Arceo et al. 2001	Primary Literature
Atlantic	Tropical Atlantic	1997-2010	15	11	2017	Arias-Gonzalez et al. 2017	Primary Literature
Atlantic	Tropical Atlantic	1993-2000	5	6	2000	Aronson and Precht 2000	Primary Literature
Atlantic	Tropical Atlantic	1996-2001	5	9	2002	Aronson et al. 2002	Primary Literature
Indian	Western Indo-Pacific	2002	N/A	12	2005	Arthur et al. 2005	Primary Literature
Indian	Western Indo-Pacific	2000-2003	8.5	22	2006	Arthur et al. 2006	Primary Literature
Red Sea	Western Indo-Pacific	2016	2.5-8.8	8	2019	Atta et al. 2019	Primary Literature
Pacific	Central Indo-Pacific	1992-2020	N/A	4054		Australian Institute of Marine Science (AIMS)	Database
Indian	Central Indo-Pacific	2006	0.4-7.1	26	2011	Bancroft 2011	Primary Literature
Atlantic	Tropical Atlantic	2010-2011	N/A	4	2012	Bastidas et al. 2012	Primary Literature
Pacific	Central Indo-Pacific	2006	5-9.75	7	2012	Becira et al. 2012	Primary Literature
Atlantic	Tropical Atlantic	2006-2008	0.1	4	2011	Belford and Phillip 2001	Primary Literature
Indian	Western Indo-Pacific	1997	5.5	11	2001	Bergman and Ohman 2001	Primary Literature
Pacific	Eastern Indo-Pacific	1995	3.1-12.2	31	2003	Birkeland et al. 2003	Primary Literature
Pacific	Central Indo-Pacific	1998	3-10	18	2001	Bruno et al. 2001	Primary Literature
The Gulf	Western Indo-Pacific	2017-2018	4	24	2019	Burt et al. 2019	Primary Literature
Pacific	Central Indo-Pacific	2006-2010	2-10	18	2012	Cabaitan et al. 2012	Primary Literature

Atlantic	Tropical Atlantic	2008	7.8-20.5	5	2020	Cáceres et al. 2020	Primary Literature
Indian	Central Indo-Pacific	2005-2009	4-9	24	2011	Ceccarelli et al. 2011	Primary Literature
Atlantic	Tropical Atlantic	1996-1998	4.2-11.9	10	2001	Chiappone et al. 2001	Primary Literature
Atlantic	Tropical Atlantic	1978-2016	N/A	65	2020	Contreras-Silva et al. 2020	Primary Literature
Atlantic, Pacific	Tropical Atlantic, Tropical Eastern Pacific	1994-2005	5-12	25	2010	Cortes et al. 2010	Primary Literature
Atlantic	Tropical Atlantic	2005-2008	N/A	4	2010	Crabbe 2010	Primary Literature
Pacific	Central Indo-Pacific	2007	N/A	7	2012	Dadhich et al. 2012	Primary Literature
Indian	Central Indo-Pacific	2010-2011	3	8	2013	Depczynski et al. 2013	Primary Literature
Pacific	Eastern Indo-Pacific	2004-2009	3-10	21	2010	Donner et al. 2010	Primary Literature
Atlantic	Tropical Atlantic	1992-1998	N/A	42	2002	Edmunds 2002	Primary Literature
Atlantic	Tropical Atlantic	1987-2011	9	56	2013	Edmunds 2013	Primary Literature
Atlantic, Pacific	Tropical Atlantic, Temperate Northern Atlantic, Central Indo-Pacific, Eastern Indo-Pacific	2012-2017	0.3-32.3	4246		NOAA NCRMP	Database
Pacific	Central Indo-Pacific	2018	2-10.5	9	2019	Fazekas 2019	Primary Literature
Pacific	Eastern Indo-Pacific	2009	9	1	2012	Fenner 2012	Primary Literature
Atlantic	Tropical Atlantic	1996-2017	N/A	1671		Florida CREMP	Database
The Gulf, Indian	Western Indo-Pacific	2006-2009	2.7-8.1	37	2012	Foster et al. 2012	Primary Literature
Red Sea	Western Indo-Pacific	2010	5-15	24	2013	Furby et al. 2013	Primary Literature

Atlantic	Tropical Atlantic	1997-1998	10	3	2001	Garza-Perez and Arias-González 2001	Primary Literature
Atlantic	Tropical Atlantic	1986	6-18	3	1989	Gates 1989	Primary Literature
Pacific	Central Indo-Pacific	2012	5.3-8.3	12	2014	Gonzales and Gonzales 2014	Primary Literature
Atlantic	Tropical Atlantic	2005-2017	N/A	17	2020	Gonzalez-Barrios et al. 2020	Primary Literature
Pacific, Atlantic, Indian	Central Indo-Pacific, Eastern Indo-Pacific, Tropical Atlantic	2012-2018	10	860	2019	González-Rivero et al. 2019	Primary Literature
Indian	Western Indo-Pacific	1994-2005	N/A	25	2007	Graham et al. 2007	Primary Literature
Indian	Western Indo-Pacific	1994-2014	N/A	54	2015	Graham et al. 2015	Primary Literature
Indian	Western Indo-Pacific	2005-2014	N/A	36	2020	Graham et al. 2020	Primary Literature
Indian	Central Indo-Pacific	2000	1.5-5	17	2000	Grubba and Cary 2000	Primary Literature
Pacific	Central Indo-Pacific	1987-2012	3.5-6.5	190	2016	Guest et al. 2016	Primary Literature
Pacific	Tropical Eastern Pacific	1985-1986	0.8-11	36	1990	Guzman et al. 1990	Primary Literature
Pacific	Central Indo-Pacific	2013	5	9	2015	Hadi et al. 2015	Primary Literature
Indian	Western Indo-Pacific	1999-2005	15	4	2008	Hagan et al. 2008	Primary Literature
Indian	Western Indo-Pacific	2005	10	1	2010	Hagan et al. 2010	Primary Literature
Indian	Western Indo-Pacific	2017	5	4	2019	Harahap et al. 2019	Primary Literature
Pacific	Central Indo-Pacific	1996	1-18	7	1997	Harborne et al. 1997	Primary Literature
Pacific	Central Indo-Pacific	2001	1-20	22	2001	Harborne et al. 2001	Primary Literature
Indian	Western Indo-Pacific	2005	6.5-21	10	2006	Harding et al. 2006	Primary Literature
Indian	Western Indo-Pacific	2006	1.3	3	2007	Hardman et al. 2007	Primary Literature

Indian	Central Indo-Pacific	2009-2015	N/A	31	2019	Haywood et al. 2019	Primary Literature
Atlantic	Tropical Atlantic	2003-2006	6-30	12	2007	Herzlieb et al. 2007	Primary Literature
The Gulf, Atlantic, Indian, Pacific, Red Sea	Central Indo-Pacific, Eastern Indo-Pacific, Temperate Australasia, Temperate Northern Pacific, Temperate Southern Africa, Tropical Atlantic, Tropical Eastern Pacific, Western Indo-Pacific	1997-2020	0.1-24	14334		Reef Check	Database
Pacific	Central Indo-Pacific	1995-2009	N/A	14	2013	Hongo and Yamano 2013	Primary Literature
Pacific	Central Indo-Pacific	2010-2011	3-8	20	2014	Huang et al. 2014	Primary Literature
Atlantic	Tropical Atlantic	1977-1993	N/A	18	1994	Hughes 1994	Primary Literature
Atlantic	Tropical Atlantic	1977-1993	7	6	1999	Hughes and Connell 1999	Primary Literature
Atlantic	Tropical Atlantic	1977-1993	35	17	2000	Hughes and Tanner 2000	Primary Literature
Atlantic	Tropical Atlantic	1995-2004	7	3	2006	Idjadi et al. 2006	Primary Literature
Indian	Western Indo-Pacific	2003-2013	1.5-3.5	4	2013	Jameel 2013	Primary Literature
Pacific	Central Indo-Pacific	1996-2003	N/A	13	2004	Jones et al. 2004	Primary Literature
Pacific	Eastern Indo-Pacific	2002	2.1-14.9	17	2006	Kenyon et al. 2006	Primary Literature
Red Sea	Western Indo-Pacific	2013	2-10	18	2017	Khalil et al. 2017	Primary Literature
Pacific	Central Indo-Pacific	2002	3-7.5	20	2002	Koh et al. 2002	Primary Literature
Indian	Western Indo-Pacific	2003	3.6-12	15	2003	Koh et al. 2003	Primary Literature

Pacific	Central Indo-Pacific	1985-2010	8-14	9	2012	Kuo et al. 2012	Primary Literature
Indian	Western Indo-Pacific	2004	1-6	13	2006	Lambo and Ormond 2006	Primary Literature
Indian	Western Indo-Pacific	2006-2020	7	10	2021	Lange et al. 2021	Primary Literature
Indian	Western Indo-Pacific	1994-2005	N/A	8	2007	Ledlie et al. 2007	Primary Literature
Pacific	Eastern Indo-Pacific	2012	2.5	8	2016	Lewis 2016	Primary Literature
Atlantic	Tropical Atlantic	1977-1982	0.5-30	6	1987	Liddell and Ohlhorst 1987	Primary Literature
Atlantic	Tropical Atlantic	2004	8.5	1	2007	Lirman et al. 2007	Primary Literature
Indian	Western Indo-Pacific	2004	4-13	26	2004	Loh et al. 2004	Primary Literature
Indian	Central Indo-Pacific	2006	3	17	2007	Long 2007	Primary Literature
Pacific	Eastern Indo-Pacific	2004	19.3-25	5	2004	Lovell 2004	Primary Literature
Pacific	Central Indo-Pacific	1999-2007	N/A	18	2008	Lovell and Sykes 2008	Primary Literature
Indian	Western Indo-Pacific	2003	1	2	2007	Mangubhai et al. 2007	Primary Literature
Indian	Western Indo-Pacific	1994-1996	1-1.5	6	1996	McClanahan et al. 1996	Primary Literature
Indian	Western Indo-Pacific	1996	1.5	12	1999	McClanahan et al. 1999	Primary Literature
Indian	Western Indo-Pacific	1996-2010	3.75	14	2015	McClanahan et al. 2015	Primary Literature
Indian	Western Indo-Pacific	2016	2-5.5	18	2019	McClanahan et al. 2019	Primary Literature
Atlantic	Tropical Atlantic	1997	15.5	12	2001	McField et al. 2001	Primary Literature
Indian	Western Indo-Pacific	2002	5-14	46	2003	McKenna and Allen 2003	Primary Literature
Atlantic	Tropical Atlantic	2005-2007	N/A	30	2009	Miller et al. 2009	Primary Literature
Red Sea	Western Indo-Pacific	2009	3-8	7	2012	Mohammed 2012	Primary Literature

Red Sea	Western Indo-Pacific	2004	6-7	3	2005	Mohamed and Mohamed 2005	Primary Literature
Red Sea	Western Indo-Pacific	2015	N/A	11	2018	Monroe et al. 2018	Primary Literature
Indian	Western Indo-Pacific	2015-2019	5	50	2020	Montefalcone et al. 2020	Primary Literature
Indian	Western Indo-Pacific	2000	2-25.5	11	2002	Motta et al. 2002	Primary Literature
Pacific	Central Indo-Pacific	2018	3.5-10.5	6	2020	Mujiyanto et al. 2020	Primary Literature
Red Sea	Western Indo-Pacific	2010-2013	7	9	2015	Naumann et al. 2015	Primary Literature
Atlantic	Tropical Atlantic	1994-1998	16	7	2000	Ostrander et al. 2000	Primary Literature
Indian	Western Indo-Pacific	2014	12-21	5	2014	Pereira and Fernandes 2014	Primary Literature
Indian	Western Indo-Pacific	2000	5	2	2004	Pereira and Gonçalves 2004	Primary Literature
Atlantic	Tropical Atlantic	2009	10-20	6	2011	Pereira-Filho et al. 2011	Primary Literature
Pacific	Central Indo-Pacific	1995-1998	7.8-5	40	1999	Poosuwan 1999	Primary Literature
Indian, Pacific	Western Indo-Pacific	2005	3	3	2006	Raj et al. 2006	Primary Literature
Indian	Western Indo-Pacific	1994	8-22	2	1997	Rajasuriya et al. 1997	Primary Literature
Atlantic	Tropical Atlantic	2007	4	1	2010	Ramos et al. 2010	Primary Literature
Pacific, Indian	Central Indo-Pacific, Western Indo-Pacific	2001-2016	5-10	323		ReefCloud	Database
The Gulf	Western Indo-Pacific	2007	6	13	2010	Rezai et al. 2010	Primary Literature
Pacific	Central Indo-Pacific, Temperate Northern Pacific	2011	5	25	2016	Ribas-Deulofeu et al. 2016	Primary Literature
Indian	Central Indo-Paciifc	2009-2014	0-14	101	2018	Richards et al. 2018	Primary Literature

Indian	Central Indo-Pacific	2016	0-13.5	11	2019	Richards et al. 2019	Primary Literature
Indian	Central Indo-Pacific	2008-2013	3-6	35	2016	Ridgway et al. 2016	Primary Literature
Atlantic	Tropical Atlantic	1996-2005	12	15	2009	Riegl et al. 2009	Primary Literature
Pacific	Central Indo-Pacific	2002-2018	N/A	332	2021	Roelfsema et al. 2021	Primary Literature
Atlantic	Tropical Atlantic	1989-2002	7.6-14.4	27	2006	Rogers and Miller 2006	Primary Literature
Atlantic	Tropical Atlantic	1979	1-6	3	1982	Rützler and Macintyre 1982	Primary Literature
Pacific	Central Indo-Pacific	2007	6	4	2013	Salinas-de-León et al. 2013	Primary Literature
Atlantic	Tropical Atlantic	2015-2016	2.8-18	22	2019	Sánchez et al. 2019	Primary Literature
Pacific	Central Indo-Pacific	2010-2015	N/A	15	2017	Saptarini et al. 2017	Primary Literature
Pacific	Central Indo-Pacific	2007	4-19	10	2008	Scaps and Runtukahu 2008	Primary Literature
Pacific	Eastern Indo-Pacific	2002-2006	10.7-15.7	6	2011	Schopmeyer et al. 2011	Primary Literature
Atlantic	Tropical Atlantic	1999	3.6-4.8	2	2011	Segal and Castro 2011	Primary Literature
Indian	Western Indo-Pacific	2006	5-25	24	2008	Sheppard et al. 2008	Primary Literature
Pacific	Central Indo-Pacific	2011	7.5	9	2013	Siringoringo and Hadi 2013	Primary Literature
Pacific	Central Indo-Pacific	2012	7.5	6	2014	Siringoringo and Hadi 2014	Primary Literature
Indian	Central Indo-Pacific	1996-2004	9	42	2008	Smith et al. 2008	Primary Literature
Indian	Western Indo-Pacific	1998	15	4	2000	Spencer et al. 2000	Primary Literature
Pacific	Eastern Indo-Pacific	2008-2010	1	8	2012	Stender 2012	Primary Literature
Atlantic	Tropical Atlantic	1999	N/A	14	2003	Steneck and Lang 2003	Primary Literature

Atlantic	Tropical Atlantic	2004-2017	0.9-11.9	306	2019	Steneck et al. 2019	Primary Literature
Indian	Western Indo-Pacific	1999-2003	10-20	8	2005	Stobart et al. 2005	Primary Literature
Pacific	Central Indo-Pacific	2004	3-6	16	2004	Tanzil and Chou 20014	Primary Literature
Atlantic	Tropical Atlantic	2006-2018	N/A	55	2021	Teixeria et al. 2021	Primary Literature
Indian	Western Indo-Pacific	2010-2011	4	15	2012	Tkachenko 2012	Primary Literature
Pacific	Central Indo-Pacific	2007	3.5-9	8	2010	Tkachenko and Soong 2010	Primary Literature
Pacific	Central Indo-Pacific	2013-2019	4	27	2021	Tkachenko et al. 2021	Primary Literature
Pacific	Central Indo-Pacific	2008-2017	N/A	10	2018	Torda et al. 2018	Primary Literature
Pacific	Eastern Indo-Pacific	1979-2009	2.5-20	50	2011	Trapon et al. 2011	Primary Literature
Pacific	Central Indo-Pacific	1995-2000	N/A	57	2000	Tuan 2000	Primary Literature
Red Sea	Western Indo-Pacific	2013	10	4	2016	van Hoytema et al. 2016	Primary Literature
Pacific	Central Indo-Pacific	1997-2010	2	7	2011	van Woesik et al. 2011	Primary Literature
Pacific	Tropical Eastern Pacific	1998	N/A	13	2001	Vargas-Angel et al. 2001	Primary Literature
Pacific	Eastern Indo-Pacific	2014-2015	2.2-9.9	12	2017	Vargas-Ángel et al. 2017	Primary Literature
Pacific	Eastern Indo-Pacific	2018	3-24	221	2019	Vargas-Ángel et al. 2019	Primary Literature
Pacific	Central Indo-Pacific	2012	4-9	55	2015	Waheed et al. 2015	Primary Literature
Pacific	Central Indo-Pacific	2002	1-20	22	2002	Walker et al. 2002	Primary Literature
Atlantic	Tropical Atlantic	2009	2-6	11	2011	Wallace 2011	Primary Literature
Pacific	Central Indo-Pacific	1992-2008	5-11.5	27	2008	White et al. 2008	Primary Literature
Pacific	Eastern Indo-Pacific	2008	2.5-9.5	12	2010	Williams 2010	Primary Literature

Pacific	Central Indo-Pacific	2011	3-10	2	2016	Wiyanto 2016	Primary Literature
Pacific	Central Indo-Pacific	1980-2013	N/A	22	2018	Wong et al. 2018	Primary Literature
Pacific	Central Indo-Pacific	2013	2-15	36	2015	Yang et al. 2015	Primary Literature
Pacific	Central Indo-Pacific	2016	3-6	20	2019	Yuanike et al. 2019	Primary Literature
